# Reminiscences of Prof. Gaillard; Substitute for Tracheotomy

**Published:** 1886-07

**Authors:** W. Neal Watt


### A REMINISCENCE OF GAILLARD—SUBSTITUTES FOR TRACHEOTOMY.

(For Daniel's Texas Medical Journal.)

When I was a first course student of Medicine, at the Louisville MedicalCollege, our beloved and justly distinguished Prof. E. S. Gaillard, (whose lectures were always replete with facts of practical importance) gave the members of his class many hints which have doubtless since proved available in trying situations.

In these days of the world's advance, when malignant affections of the throat,—diphtheritic, and croupous, seem to be on the increase and their fatality also, much greater than formerly, the physician is often brought face to face with that forlorn hope —laryngotomy or tracheotomy ; and having recently encountered a difficult case of croup, my memory has reverted to the period when I sat an attentive listener to the ripe advice that fell from the lips of that eloquent teacher. Indeed—through all the vicissitudes of my student life—notwithstanding I have been under the instruction of some of the most illustrious men of the day in America—the truths and precepts inculcated by that grand and heroic one-armed Confederate soldier most impressed me, and shall ever remain the choicest portion of my student experience.

The case referred to was like most of them in my experience, uncompromising in the onset, rapid, the supervention of alarming dispnœa prompt. and the approach of the end fearfully obvious. What shall I do? Perform laryngotomy or tracheotomy? No! oh, no! I tried the knife once, and he who has used it once in these affections will be loth to do so again ; for croup loves a wound as dearly as does its twin sister, diphtheria ; and oh!—the hemorrhage and its consequences! What then must we do—can we see our patient die without an effort on our part to save it? Here I remembered suggestions received from Dr. Gaillard. "In these distressing conditions" said he, "after deciding to operate, we must select the operation freest from danger, primary and remote ***. My plan has been to use, instead of actual incision, attending which there is always much hæmorrhage, the ordinary trocar and canula. The puncture must be made with something like a quick thrust, and semi-rotation, and moderate and equal pressure ; after perforation of the inter-cartilaginous space selected,—carefully avoiding implicating important vessels, remove the trocar, as in paracentisis,—and respiration will readily take place through the improvised channel, to the relief of and complete mitigation of all embarrassing symptoms and to the joy of anxious friends."

"Another good plan to avoid hemorrhage in tracheotomy is to use an ordinary triangular pointed lancet heated to a white heat, puncturing with it the membranes at the chosen site of operation. This may seem barbarous to the unitiated, but it must be remembered that in the semi-asphyxiated state sensibility is greatly reduced—the thorough impregnation of the system with carbonic acid gas effectually obtunds feeling and establishes, to all intents and purposes a state of local anæsthesia. The local effect of the cutting in question is beyond doubt highly beneficial, all morbid organization surrounding the point of entrance of the heated lancet is completely destroyed and the raw surface is not so apt to become affected, nor occluded, clogged by the accumulation of disease products."

Either of these modes of operating in croup or diphtheria has advantages over laryngotomy or tracheotomy as ordinarily performed.

Dr. Gaillard also suggested in this connection the use of a small elastic catheter, introduced into the larynx per os, in croup or diphtheria—a means sometimes utilized with marked success. [He should really have the credit for suggesting "intubation of the larynx." Ed.]

In presenting these few reminiscences of college life and of my old master to the consideration of my professional brethren, let it be understood that I claim nothing for myself, no originality in connection with the subject, nor for the distinguished Professor. These ideas may have originated with him and they may not. I only know they were new to me and have proved useful in my hands. If this last assurance will induce some timid brother who instinctively shrinks from the horrors of tracheotomy to undertake the relief of some little sufferer by the plan here indicated, I shall be compensated for this hastily written article. W. Neal Watt, M. D.

